# Local Vascularization during Orthodontic Tooth Movement in a Split Mouth Rat Model—A MRI Study

**DOI:** 10.3390/biomedicines8120632

**Published:** 2020-12-19

**Authors:** Peter Proff, Agnes Schröder, Lisa Seyler, Franziska Wolf, Yüksel Korkmaz, Tobias Bäuerle, Lina Gölz, Christian Kirschneck

**Affiliations:** 1 Department of Orthodontics, University Hospital Regensburg, 93053 Regensburg, Germany; agnes.schroeder@ukr.de (A.S.); franziska2.wolf@stud.uni-regensburg.de (F.W.); christian.kirschneck@ukr.de (C.K.); 2 Department of Radiology, University Clinic, Friedrich-Alexander-University Erlangen-Nuremberg, 91054 Erlangen, Germany; lisa.seyler@uk-erlangen.de (L.S.); tobias.baeuerle@uk-erlangen.de (T.B.); 3 Department of Periodontology and Operative Dentistry, University of Mainz, 55131 Mainz, Germany; Yueksel.Korkmaz@unimedizin-mainz.de; 4 Department of Orthodontics, University Clinic, Friedrich-Alexander-University Erlangen-Nuremberg, 91054 Erlangen, Germany; Lina.Goelz@uk-erlangen.de

**Keywords:** magnetic resonance imaging, orthodontic tooth movement, vascularization

## Abstract

Orthodontic tooth movement to therapeutically align malpositioned teeth is supposed to impact blood flow in the surrounding tissues. Here, we evaluated actual vascularization in the tension area of the periodontal ligament during experimental tooth movement in rats (*N* = 8) with magnetic resonance imaging (MRI). We inserted an elastic band between the left upper first and the second rat molar; the right side was not treated and served as control. After four days of tooth movement, we recorded T1-weighted morphologic and dynamic-contrast-enhanced MRI sequences with an animal-specific 7 Tesla MRI to assess of local vascularization. Furthermore, we quantified osteoclasts and monocytes in the periodontal ligament, which are crucial for orthodontic tooth movement, root resorptions as undesirable side effects, as well as the extent of tooth movement using paraffine histology and micro-CT analysis. Data were tested for normal distribution with Shapiro–Wilk tests followed by either a two-tailed paired *t*-test or a Wilcoxon matched-pairs signed rank test. Significant orthodontic tooth movement was induced within the four days of treatment, as evidenced by increased osteoclast and monocyte activity in the periodontal ligament as well as by µCT analysis. Contrast enhancement was increased at the orthodontically-treated side distally of the moved upper first left molar, indicating increased vascularization at the tension side of the periodontal ligament. Accordingly, we detected reduced time-to-peak and washout rates. Our study using MRI to directly assess local vascularization thus seems to confirm the hypothesis that perfusion is enhanced in tension zones of the periodontal ligament during orthodontic tooth movement.

## 1. Introduction

According to the pressure–tension theory of orthodontic tooth movement, treatment with fixed or removable orthodontic appliances results in the development of compressed and stretched areas within the periodontal tissue with alveolar bone resorption at compression and alveolar bone formation at tension areas [1,2,3], which could last up to 6 months into the retention phase [4]. These mechanical stimuli are recognized by several cell types in the tooth surrounding tissues, including periodontal ligament fibroblasts, osteoblasts, osteocytes, and immune cells via mechanosensitive channels and receptors leading to adapted expression of cellular mediators [2,3].

It was shown before that periodontal ligament fibroblasts react to compressive forces and tensile loading with a modified secretion of inflammatory factors such as prostaglandin E2 and interleukin-6 [5,6,7,8]. This results in elevated expression levels of receptor activator of nuclear factor kappa b ligand (RANKL) upon compressive force treatment in periodontal ligament fibroblasts [6,8,9]. The interaction of RANKL and its decoy receptor osteoprotegerin (OPG), however, is essential for the differentiation of osteoclast progenitor cells to bone-resorbing osteoclasts, thereby regulating the resorption of alveolar bone and finally orthodontic tooth movement [10,11,12]. Furthermore the RANKL/OPG system is strongly involved in a cross-regulation of the bone and immune system [11]. RANKL was shown to be expressed by several cell types, including T-cells [13] and keratinocytes [14], and high levels of RANKL were associated with type-2 diabetes mellitus, for example [15]. Furthermore, it was shown that protein structure and periodontal fibers in the periodontal ligament (PDL) are modified after different timepoints of orthodontic force application [16]. Recently, it has been shown that osteoblast activity in tension areas is heavily dependent on osteoclast activity via crosstalk according to the biphasic theory of tooth movement. This means that osteoclastic processes precede osteoblastic activity during the molecular processes, enabling orthodontic tooth movement [3,17].

Next to enhanced secretion of cytokines and mediators via mechanically affected cells, orthodontic treatment may also influence the blood flow in the periodontal tissues. It is known that periodontal ligament fibroblasts and macrophages increase the expression of vascular endothelial growth factor (VEGF) in vitro after pressure application [6,18]. VEGF is an essential growth factor involved in angiogenesis and vasculogenesis [19]. The main transcriptional regulator of VEGF expression is the transcription factor hypoxia inducible factor-1-alpha (HIF1α) [20]. Orthodontic treatment may cause hyopoxic conditions in the periodontal ligament due to compressed blood vessels promoting HIF1α stabilization. Recent studies showed that macrophages stabilize HIF1α only due to deprived oxygen supply and not in response to pressure application [21]. In contrast, periodontal ligament fibroblasts can also stabilize HIF1α by mechanical strain itself independent of surrounding oxygen levels [22], indicating a supporting role of pressure application on angiogenesis.

To date, it is still unclear, how orthodontic force application on teeth affects local perfusion in the periodontal tissue surrounding the tooth. The null hypothesis of this study was that orthodontic tooth movement has no effect on vascularization in the periodontal ligament. To unravel this question, we performed a study in a rat model using magnetic resonance imaging (MRI) to detect vascularization within the periodontal ligament of an orthodontically moved upper rat molar. As we used only a short protocol of four days, we additionally checked for tartrate-resistant acid phosphatase (TRAP^+^) and CD68^+^ cells (osteoclasts/macrophages) via histology as well as parameters of orthodontic tooth movement via micro-CT analysis.

## 2. Materials and Methods

### 2.1. Animal Experiment

Eight male Fischer 344 rats (Rattus norvegicus Berkenhout, Charles River Laboraties, Sulzfeld, Germany) aged 7 weeks were included in this study, which was approved by the responsible authorities and performed in compliance with the German Animal Protection Act (approval ID: 55.2.2-2532.2-987-14, 13 November 2019, Government of Lower Palatinate, Germany). Sample size was determined based on the volume of increased contrast media uptake on T1-weighted morphologic images observed in a pilot study (data not shown, α = 0.05, β = 0.2). We defined termination criteria and monitored animal welfare daily. The rats were kept in a conventional animal laboratory at the University of Erlangen-Nuremberg (Preclinical Experimental Animal Centre PETZ) and had free access to a standard rat diet (V1535, ssniff, Soest, Germany) and to tap water [23,24,25]. Four days after onset of tooth movement and after MRI measurements, rats were killed according to legal guidelines.

### 2.2. Orthodontic Tooth Movement

After twelve days of acclimatization, rats were narcotized by injection of 6 mg xylazine and 90 mg ketamine per kg body weight [23,25,26,27]. An orthodontic elastic band (774-200-01, Dentaurum, Ispringen, Germany) was inserted in the approximal space between the first and the second left molar of the rat upper jaw for four days [25], whereas the contralateral jaw side remained untreated. Expansion of the compressed elastic band after insertion provoked an anterior experimental tooth movement of the first upper left molar simulating orthodontic treatment.

### 2.3. T1-Weighted Morphologic and Dynamic-Contrast-Enhanced (DCE) MRI

**Figure 1:**
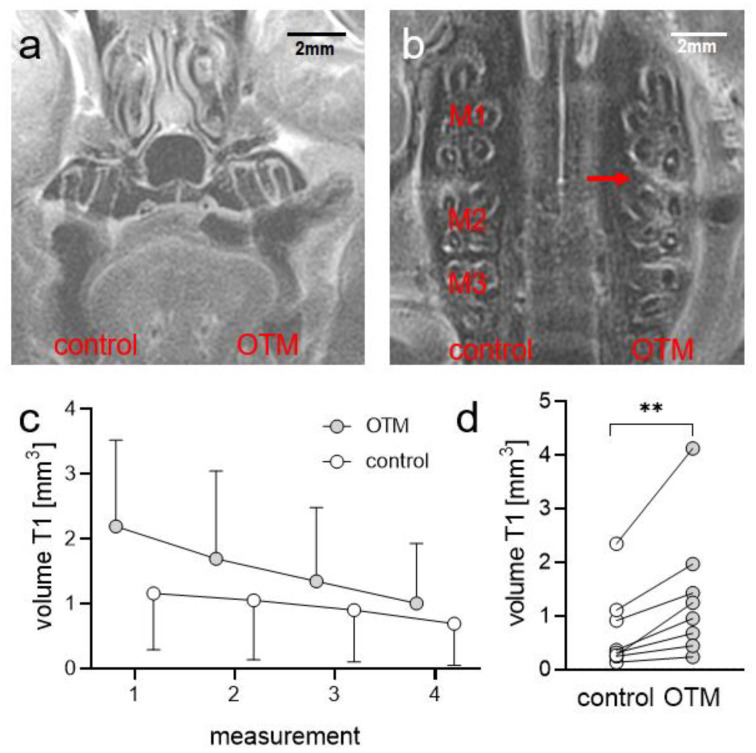
Representative magnetic resonance images. Frontal view (**a**) and axial view (**b**). Kinetics of T1-weighted MR images ((**c**); T1 volume distally of the moved upper first left molar, *n* = 4). Volume of increased contrast media uptake on T1-weighted morphologic images ((**d**); *n* = 8). Red arrow: increased contrast media uptake in T1-weighted MR image at the OTM side. OTM = orthodontic tooth movement, M1/2/3 = first/second/third upper molar. ** *p* < 0.01.

After four days of orthodontic tooth movement (OTM), rats were anesthetized with 4% isoflurane and maintained at 1.5% isoflurane. Heads were fixed in a rat brain magnetic resonance tomography (MRT) surface coil and scanned on a preclinical 7T MRI Scanner (Figure 1, ClinScan 70/30, Bruker, Ettlingen, Germany). Respiration was monitored by a pressure sensor (Small Animal Monitoring and Gating System Model1030; SA Instruments, Inc., Stony Brook, NY, USA) and kept constant by adjusting isoflurone inhalation if needed during the entire imaging procedure. Additionally, the body temperature was kept constant employing a heating bed for the animals. The periodontal region of the first upper left and right molars was analyzed using a conventional gadolinium-based contrast agent on T1-weighted morphologic images. For this purpose, a T1-weighted spin echo sequence (TR: 600 ms, TE: 10 ms, voxel size: 0.078 × 0.078 × 0.7 mm, acquisition time: 12:05 min) was run (Table S1). In addition to T1-weighted morphologic MRI datasets, we performed T1-weighted functional dynamic-contrast-enhanced (DCE) MRI using a fast low-angle shot (FLASH) sequence with the following parameters: TR: 2.92 ms, TE: 0.88 ms, flip angle: 25°, voxel size: 0.182 × 0.182 × 0.7 mm, acquisition time: 12 min 18 s and 100 measurements. After running the DCE sequence, the above-mentioned T1-weighted morphologic MRI sequence was repeated, and for determination of the enhancing volume after administration of the contrast agent, T1-weighted morphological images were segmented using a threshold of 100 for signal intensity. DCE-MRI resulted in the semi-quantitative parameters area under the curve (AUC), peak enhancement (PE), time to peak (TTP), and washout (WO).

### 2.4. Preparation of Paraffin Sections for Histology

After MRI measurements upper jaws of rats were stored in 5% formaldehyde overnight at 4 °C and were transferred to 0.1% formaldehyde until µCT measurements were performed. They were split into control and orthodontically treated sides. Afterwards, they incubated in 10% Tris-buffered ethylene diamine tetra-acetic solution (pH 7.4) at room temperature for eight weeks to demineralize the alveolar bone [23,24,25]. Both sides were embedded in paraffin and cut in sagittal-oblique sections of 5 µm with a rotating microtome (HM350, Microm International, Dreieich, Germany). They were mounted onto Superfrost glass slides to avoid detachment during staining procedures.

### 2.5. Hematoxylin and Eosin (HE) Staining for Evaluation of Root Resorptions

Prior to staining, sections incubated for 30 min at 60 °C followed by incubation for 20 min in xylene (9713.2, Carl Roth, Karlsruhe, Germany). After hydrogenation by a descending alcohol series, they were stained with Mayer hematoxylin solution (1.07961.0500, Sigma Aldrich, St. Louis, MO, USA) for 10 min. After 5 min under running warm water, sections were counterstained for one minute with 0.5% eosin G solution (X883.2, Carl Roth, Karlsruhe, Germany). Again, they were rinsed with warm water and dehydrated by the ascending series of alcohol. After 20 min in xylene, they were covered using entellan (1.07961.0500, Merck, Darmstadt, Germany). The stained histological sections were digitized using an Olympus IX50 microscope (Hamburg, Germany) at 100 fold magnification, and evaluation of root resorptions at the disto-buccal tooth root of the moved first molar in relation to the respective root surface area was assessed with ImageJ (Ver.1.4.7, National Institutes of Health, Bethesda, MD, USA), as described before [16,19].

### 2.6. Tartrate-Resistant Acid Phosphatase (TRAP) Staining for Evaluation of Osteoclasts

Paraffin sections were incubated overnight at 37 °C and hydrogenated by a descending alcohol series. For the TRAP buffer, we dissolved 1.64 g sodium acetate (6773.1, Carl Roth, Karlsruhe, Germany) and 23 g di-sodium tartrate dihydrate (T110.1, Carl Roth, Karlsruhe, Germany) in 500 mL of H_2_O_dd_ and adjusted pH to 5.0 using HCl. Slides were incubated for 10 min at room temperature in this TRAP buffer. For the staining solution, we mixed 40 mg Naphtol AS-MX Phosphate Disodium Salt (N5000, Sigma Aldrich, St. Louis, MO, USA), 4 mL *N*,*N*-dimethylformamide (D4551, Sigma Aldrich, St. Louis, MO, USA), 240 mg Fast Red Violet LB Salt (F3381, Sigma Aldrich, St. Louis, MO, USA), and 2 mL Triton X-100 (T9284, Sigma Aldrich, St. Louis, MO, USA) with 200 mL TRAP buffer and incubated the slides for two hours at 37 °C, followed by counterstaining with filtered Mayer’s hematoxylin solution (51275, Sigma Aldrich, St. Louis, MO, USA) for 3 min at room temperature. The stained histological sections were covered with Aquatex (1085620050, Merck, Darmstad, Germany) and digitized using an Olympus IX50 microscope (Hamburg, Germany). Evaluation of TRAP^+^ area within the periodontal ligament of the disto-buccal tooth root of the moved first molar in relation to the respective root surface area was assessed with ImageJ (Ver.147, National Institutes of Health, Bethesda, MD, USA), as described before [16,19].

### 2.7. CD68 Staining for Evaluation of Cells of the Mononuclear Phagocyte System

Paraffin sections were incubated at 55 °C for 15 min followed by incubation in xylene (9713.2, Carl Roth, Karlsruhe, Germany) for 20 min. After hydration by a descending alcohol series, slides were incubated for 5 min in 0.01 M citrate buffer (pH 6.0) consisting of citric acid (C0759, Sigma Aldrich, St. Louis, MO, USA) and tri-sodium citrate-dihydrate (4088.3, Carl Roth, Karlsruhe, Germany) in steam of a 93 °C water bath to slowly adapt to temperature. Then, they were transferred to a cuvette filled with citrate buffer and incubated for another 35 min at 93 °C. After cooling, they were rinsed with water and Tris-buffered phosphate, including 1% Tween-20 (P9416, Sigma Aldrich, St. Louis, MO, USA) and blocked for 10 min using Dako real peroxidase blocking solution (S2023, Agilent, Santa Clara, CA, USA) followed by incubation for 40 min in 1% goat serum (G9023, Sigma Aldrich, St. Louis, MO, USA). CD68 antibody (MCA341R, BioRad, Hercules, CA, USA) was diluted 1:100 in DAKO real antibody diluent (S202230-2, Agilent, Santa Clara, CA, USA) and incubated for 16 h at 4 °C. After washing, slides were incubated with Histofine Simple Stain MAX PO (414132F, Nichirei Biosciences, Tokyo, Japan) for exactly 30 min. After washing, staining was detected with DAKO liquid DAB+ substrate chromogen system (K3467, Agilent, Santa Clara, CA, USA). Slides were washed and counterstained for 3 min with filtered Mayer’s hematoxylin solution (51275, Sigma Aldrich, St. Louis, MO, USA). Again, they were rinsed with warm water and dehydrated by an ascending series of alcohol. After 20 min in xylene, they were covered using entellan (1.07961.0500, Merck, Darmstadt, Germany) [23,24,25]. Evaluation of relative CD68^+^ cells within the periodontal ligament of the disto-buccal tooth root of the moved first molar in relation to the respective root surface area was assessed with ImageJ (Ver.147, National Institutes of Health, Bethesda, MD, USA).

### 2.8. Analysis of the µCT Data

micro-CT measurements were performed with a GE V-Tome-X S240 (GE Healthcare, Chicago, IL, USA). Upper jaws of rats were scanned with a Fast-Scan protocol (8 min, 240kV-D tube, voxel size 17 µm; magnification 44.4 times, picture number 1000; timing 500 ms; voltage 50 kV; electricity 330). We determined periodontal gap and bone loss distally and mesially of the mesial tooth root of the move upper first molar as well as tooth inclination and distance between the first and the second molar as an indicator of orthodontic tooth movement using VGL3.0 (Volume Graphics GmbH, Heidelberg, Germany), as described before [16,17].

### 2.9. Statistical Analysis

After testing for normal distribution (Shapiro–Wilk test), either two-tailed paired *t*-test or a Wilcoxon matched-pairs signed rank test was performed using GraphPad Prism version 8.4.3 for Windows (GraphPad Software, San Diego, CA, USA). The significance level was set at *p* < 0.05.

## 3. Results

### 3.1. T1-Weighted Morphologic Magnetic Resonance Imaging (MRI)

We performed T1-weighted morphologic MRI and detected increased contrast media uptake in the periodontal gap distally of the moved upper first left molar at the tension side of the periodontal ligament (Figure 1a,b). We implemented four T1-weighted measurements and detected increased volumes of contrast-avid tissue at the orthodontically-treated side (T1 volume; Figure 1c; Table S2), which was reduced with time. Direct comparison between control and orthodontically-treated side (OTM) revealed a statistically significant increase in T1 volume (*p* = 0.0078; Figure 1d; Table S3), indicating increased vascularization at the tension side of the moved molar.

### 3.2. Dynamic-Contrast-Enhanced Magnetic Resonance Imaging (MRI)

**Figure 2:**
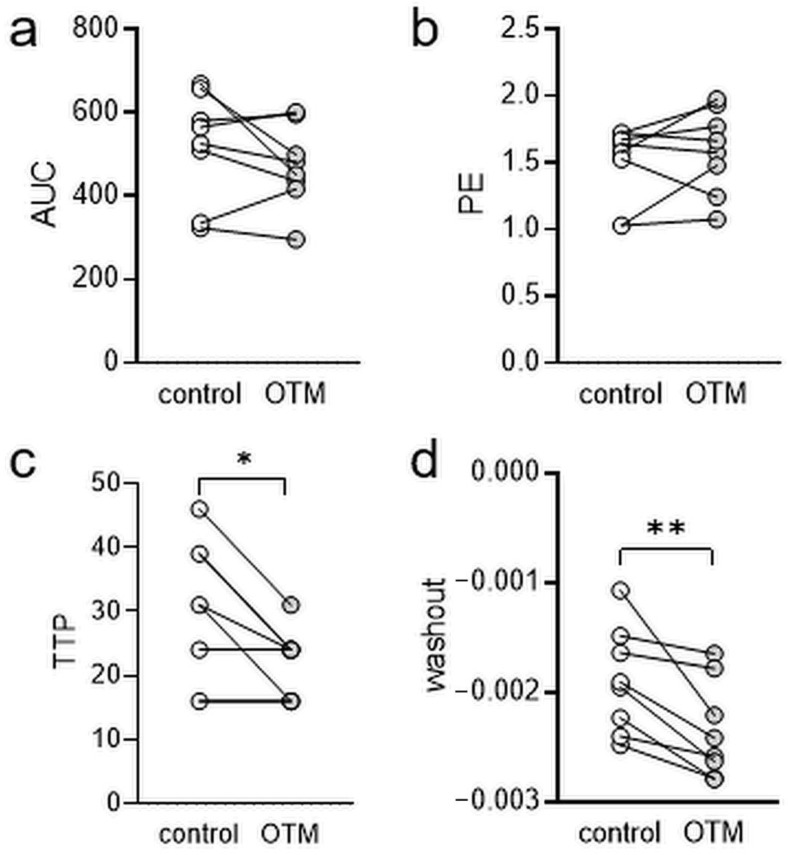
Determination of area under the curve (AUC; (**a**)), peak enhancement (PE; (**b**)), time to peak (TTP; (**c**)), and washout rate (**d**) of dynamic-contrast-enhanced MRI (*n* = 8). OTM = orthodontic tooth movement. * *p* < 0.05. ** *p* < 0.01.

Analysis of dynamic-contrast-enhanced (DCE) MRI datasets revealed no changes in signal intensity (area under the curve AUC, *p* = 0.2131; Figure 2a; Table S4) and initial peak enhancement (PE, *p* = 0.3828; Figure 2b; Table S5). PE reflects the degree of signal enhancement in the tissue after administration of the contrast agent. However, time to peak (TTP) was significantly reduced at the orthodontically treated jaw side (*p* = 0.0154; Figure 2c; Table S6). Accordingly, we found an elevated washout rate during OTM (*p* = 0.0067; Figure 2d; Table S7), indicating altered perfusion at the tension area, therefore, we rejected the null hypothesis.

### 3.3. Histological Assessment of Root Resorptions, TRAP^+^ and CD68^+^ Cells

**Figure 3:**
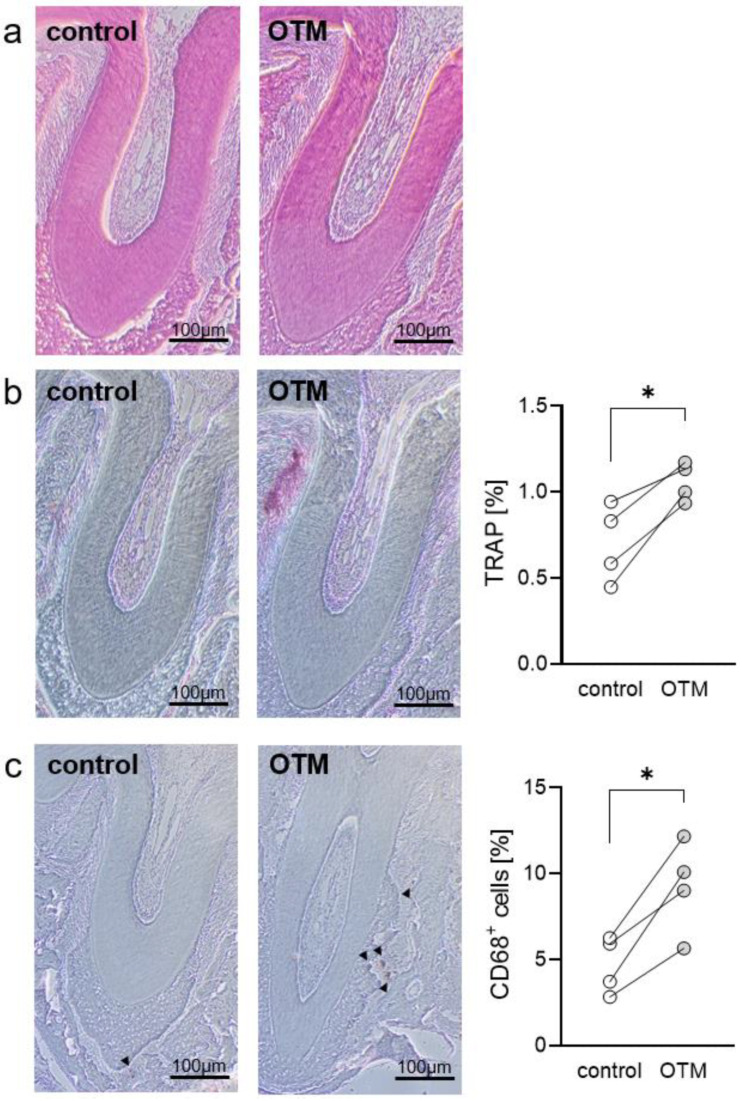
Assessment of relative root resorption (**a**), TRAP^+^ (**b**) and CD68^+^ area ((**c**), black triangles) within the periodontal ligament of the disto-buccal tooth root of the moved upper first left molar (*n* = 4). OTM = orthodontic tooth movement. * *p* < 0.05.

To confirm that orthodontic tooth movement (OTM) was successfully induced by the chosen experimental model, we evaluated osteoclast (TRAP^+^) and mononuclear phagocyte (CD68^+^) activity as well as root resorptions, which are known to be associated with and prerequisite for OTM. First, we examined root resorptions using HE-stained histological slides (Figure 3a). We detected no root resorptions on the control or on the orthodontal treated side, which may have been due to the very limited time of orthodontic treatment. However, TRAP-staining revealed significantly more TRAP^+^ cells at the orthodontically-treated side (*p* = 0.0296; Figure 3b; Table S8), indicating more osteoclastogenesis. Next, we were interested in the infiltration of cells of the mononuclear phagocyte system (MPS) and stained the sections with a CD68 antibody. We detected more CD68^+^ cells after insertion of the elastic band between the first and the second molar (*p* = 0.0161; Figure 3c; Table S9), indicating an infiltration of MPS cells.

### 3.4. Evaluation of Tooth Movement by µCT Analysis

**Figure 4:**
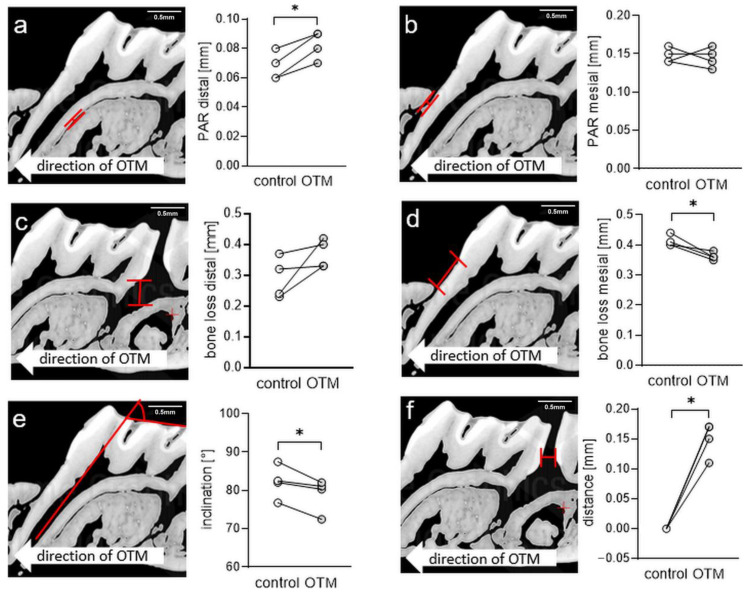
Determination of periodontal gap (**a**,**b**) and bone loss (**c**,**d**) distally and mesially of the mesial tooth root of the move upper first molar as well as tooth inclination (**e**) and distance (**f**) between the first and the second molar as an indicator of orthodontic tooth movement (*n* = 4). Red line: measured distance or angle. OTM = orthodontic tooth movement. * *p* < 0.05.

Next, we assessed the amount of orthodontic tooth movement induced by insertion of the elastic band by µCT analysis. We observed a significant increase of the periodontal gap at the distal side of the mesial tooth root of the upper first left molar after orthodontic tooth movement (*p* = 0.0138; Figure 4a; Table S10), whereas the respective mesial periodontal gap remained unchanged (*p* = 0.7888; Figure 4b; Table S10). Measurement of periodontal bone loss at the distal side of the same root revealed no changes due to orthodontic treatment (*p* = 0.1294; Figure 4c; Table S11). However, we detected a reduced bone loss mesially, which might be explained by the tipping of the first molar in anterior direction as a consequence of OTM (*p* = 0.0265; Figure 4d; Table S11). Accordingly, we measured a reduced tooth inclination at the treated side (*p* = 0.0439; Figure 4e; Table S12). Measurement of the distance between the first and the second molar, indicating total amount of tooth movement revealed a significant increase compared to the untreated control side (*p* = 0.0018; Figure 4f; Table S13).

## 4. Discussion

This study was performed to improve the insight into the role of local oxygen availability and vascularization of the tooth surrounding tissue during orthodontic force application in a short-time rat model.

After four days of tooth movement, we detected no force-induced root resorptions but an increased numbers of osteoclasts and mononuclear phagocytes, which can, on the one hand, modulate orthodontic tooth movement and, on the other hand, serve as progenitor cells for osteoclasts themselves, thus indicating that orthodontic tooth movement was successfully induced in the short-time rat model.

We furthermore observed an increased approximal distance between the moved first and the second upper left molar after four days of treatment with an elastic band. This is in line with previous studies in mice, which report orthodontic tooth movement induced by an elastic band as early as within three days and increased osteoclast numbers within seven days [28]. The other commonly employed rat model to induce OTM using a nickel-titan-coil spring [29] was not suitable for this study due to artifacts otherwise created during MRI, as we planned to perform MRI analysis with orthodontic aperture in situ. With the nickel-titan-coil spring force-induced root resorptions and increased osteoclast, numbers were reported after 14 days of treatment [24,29].

With MRI, we analyzed T1-weighted morphologic and dynamic-contrast-enhanced images. In T1-weighted morphologic images, we observed a reduction of the signal over time on both the control and the orthodontically-treated side of the jaw, indicating that the contrast agent was washed out of the local tissue over time. Of note, the volume of increased contrast media uptake at the OTM side was higher at the beginning of the measurements and over the complete investigated time, indicating that local vascularization at the treated side distally of the moved first upper left molar corresponding to the tension area of the periodontal ligament was significantly higher. Insertion of orthodontic appliances can impact on the oral environment by altering periodontal parameters [30], and tissue responses can also be affected by oral environment composition such as periodontitis [31], which might have a potential impact on results. Furthermore, a plethora of other factors such as diet and T1-weighted morphologic images display the relaxation of the nuclear spins, the alignment of which is affected by the magnetic field [32,33,34], indicating a higher accumulation of the contrast agent in the orthodontically-treated side of the jaw compared to the untreated side. Analysis of dynamic-contrast-enhanced MRI revealed no changes in AUC (area under the curve) and PE (peak enhancement) but reduced TTP (time to peak) and washout rate at the orthodontically-treated side compared to the control side, indicating an altered perfusion at the tension side of the periodontal ligament after four days of orthodontic treatment.

Several limitations of this study should be mentioned. Analysis was performed after only four days of tooth movement, and we took no MRI images before orthodontic treatment, because long-lasting MRI scanning may affect the well-being of the rats. The usage of an elastic band to induce orthodontic tooth movement does not allow a controlled force application, with forces declining over time with the decompression of the band. However, this method has been described and published before as an established means to induce orthodontic tooth movement in mice [28]. Furthermore, a higher MRI resolution would have been desirable to better differentiate and localize the vascularization changes. However, the MRI scanner used in this study is currently the MRI scanner with the highest resolution available for small animals. In the future, further developments in MRI technology may allow further insights into this matter.

Nevertheless, our findings are consistent with the current theory, which states that microcirculation is reduced at the pressure side of the periodontal tissue due to compression of the blood vessels [2,35,36] and increased at the tension side due to increased angiogenesis induced by a release of VEGF in the local tissue [2]. Inflammation is associated with an increased perfusion of the tissue [37], and orthodontic tooth movement is also described as “sterile inflammation” [2]. New insights into the molecular mechanisms underlying orthodontic tooth movement have been recently described and summarized by Proffit et al. [3] and Alikhani et al. [17], suggesting that the catabolic phase of osteoclast activity precedes the anabolic phase of alveolar bone formation, with the latter being dependent on osteoclasts via osteoclast–osteoblast crosstalk. This is in line with results from Perinetti et al., who found that the anabolic phase extends far into the retention phase after the end of active treatment, indicating a time lag of anabolic processes. The increased vascularization observed at tension areas might also be a prerequisite for local tissue remodeling and structural changes with protein structure, local biochemistry, and periodontal fibers in the PDL being modified after different timepoints of orthodontic force application, as was recently shown by Perillo et al. using advanced Micro-Raman spectroscopy [16].

In the future, advanced imaging technologies such as MRI with specific molecular markers coupled to the contrast agent (molecular MRI) may be useful to further study microcirculation within the periodontal ligament during orthodontic tooth movement and the molecular signals and cytokines involved in this process.

## 5. Conclusions

Our results using non-invasive MRI indicate that local perfusion in tension areas of the periodontal ligament is increased after four days of orthodontic tooth movement as induced by an elastic band.

## Supplementary Materials

The following are available online at https://www.mdpi.com/2227-9059/8/12/632/s1. Table S1: Sequence characteristics. Table S2: Descriptive statistics for kinetics of T1-weighted MR images. Table S3: Descriptive statistics for T1-weighted morphologic images. Table S4: Descriptive statistics for area under the curve (AUC). Table S5: Descriptive statistics for peak enhancement (PE). Table S6: Descriptive statistics for time to peak (TTP). Table S7: Descriptive statistics for washout rate. Table S8: Descriptive statistics for TRAP^+^ cells. Table S9: Descriptive statistics for CD68^+^ cells. Table S10: Descriptive statistics for periodontal gap. Table S11: Descriptive statistics for periodontal bone loss. Table S12: Descriptive statistics for inclination. Table S13: Descriptive statistics for distance between the first and second molar.
