# Supplementary material for: Local Vascularization during Orthodontic Tooth Movement in a Split Mouth Rat Model—A MRI Study
Source: Biomedicines. 2020 Dec 19;8(12):632. doi: 10.3390/biomedicines8120632 (PMC7766506; doi:10.3390/biomedicines8120632)
Supplement: Supplementary file 1 [file biomedicines-08-00632-s001.pdf]

Table S1: Sequence characteristics.

Parameter	setting
File Meta Information Group Length	194
Implementation Version Name	AYCANWSOSIRIX2
Specific Character Set	ISO_IR 100
Image Type	ORIGINAL\PRIMARY\M\ND
Modality	MR
Manufacturer	SIEMENS
StationName	CLINSCAN
Manufacturer Model Name	ClinScan
Patient ID	Kiefer
Scanning Sequence	SE
Sequence Variant	SP\OSP
Scan Options	PFP
MR Acquisition Type	2D
Sequence Name	se2d1
Angio Flag	N
Slice Thickness	0.699999988
Repetition Time	600
Echo Time	10
Number Of Averages	4
Imaging Frequency	300.419518
Imaged Nucleus	1H
Echo Numbers	1
Magnetic Field Strength	7.05568
Spacing Between Slices	0.7
Number Of Phase Encoding Steps	301
Echo Train Length	1
Percent Sampling	100
Pixel Bandwidth	205
Software Versions	syngo MR B15
Acquisition Matrix	0\448\312\0
In Plane Phase Encoding Direction	ROW
Flip Angle	90

.

Table S2: Descriptive statistics for kinetics of T1-weighted MR images

	measurement 1		measurement 2		measurement 3		measurement 4	
	control	OTM	control	OTM	control	OTM	control	OTM
Minimum	0.2650	1.248	0.2690	0.7090	0.2260	0.5340	0.2780	0.4270
25% Percentile	0.4273	1.293	0.3685	0.7518	0.2978	0.5640	0.2950	0.4410
Median	1.013	1.699	0.7840	1.210	0.6710	0.9275	0.4250	0.6090
75% Percentile	2.037	3.588	1.998	3.118	1.739	2.547	1.360	1.965
Maximum	2.346	4.127	2.363	3.644	2.042	2.995	1.645	2.375
Mean	1.159	2.193	1.050	1.693	0.9025	1.346	0.6933	1.005
Standard deviation	0.8700	1.325	0.9134	1.349	0.7986	1.137	0.6415	0.9231
error of mean	0.4350	0.6627	0.4567	0.6745	0.3993	0.5685	0.3208	0.4616
Lower 95% CI	-0.225	0.0840	-0.403	-0.453	-0.3682	-0.4633	-0.3276	-0.4639
Upper 95% CI	2.543	4.302	2.503	3.840	2.173	3.155	1.714	2.474

Table S3: Descriptive statistics for T1-weighted morphologic images.

	control	OTM
Minimum	0.137	0.235
25% Percentile	0.252	0.503
Median	0.340	1.10
75% Percentile	1.06	1.83
Maximum	2.35	4.13
Mean	0.713	1.39
Standard deviation	0.746	1.24
error of mean	0.264	0.439
Lower 95% CI	0.0889	0.348
Upper 95% CI	1.34	2.42

Table S4: Descriptive statistics for area under the curve (AUC).

	control	OTM
Minimum	0.1540	0.2990
25% Percentile	0.2670	0.5370
Median	0.3590	1.141
75% Percentile	1.062	1.834
Maximum	2.346	4.127
Mean	0.7226	1.413
Standard deviation	0.7391	1.221
error of mean	0.2613	0.4318
Lower 95% CI	0.1047	0.3921
Upper 95% CI	1.341	2.434

Table S5: Descriptive statistics for peak enhancement (PE).

	control	OTM
Minimum	1.029	1.075
25% Percentile	1.154	1.302
Median	1.608	1.619
75% Percentile	1.709	1.894
Maximum	1.723	1.974
Mean	1.490	1.589
Standard deviation	0.2919	0.3168
error of mean	0.1032	0.1120
Lower 95% CI	1.246	1.324
Upper 95% CI	1.734	1.854

Table S6: Descriptive statistics for time to peak (TTP).

	control	OTM
Minimum	16.00	16.00
25% Percentile	18.00	16.00
Median	31.02	24.01
75% Percentile	39.02	24.01
Maximum	46.03	31.02
Mean	30.27	21.88
Standard deviation	11.01	5.417
error of mean	3.894	1.915
Lower 95% CI	21.06	17.35
Upper 95% CI	39.47	26.41

Table S7: Descriptive statistics for washout rate.

	control	OTM
Minimum	-0.002482	-0.002794
25% Percentile	-0.002360	-0.002745
Median	-0.001930	-0.002501
75% Percentile	-0.001525	-0.001888
Maximum	-0.001070	-0.001648
Mean	-0.001897	-0.002356
Standard deviation	0.0004829	0.0004408
error of mean	0.0001707	0.0001559
Lower 95% CI	-0.002301	-0.002724
Upper 95% CI	-0.001493	-0.001987

Table S8: Descriptive statistics for $TRAP^+$ cells.

	control	OTM
Minimum	0.4480	0.9340
25% Percentile	0.4825	0.9503
Median	0.7085	1.066
75% Percentile	0.9150	1.162
Maximum	0.9430	1.172
Mean	0.7020	1.060
Standard deviation	0.2256	0.1118
error of mean	0.1128	0.05588
Lower 95% CI	0.3430	0.8817
Upper 95% CI	1.061	1.237

Table S9: Descriptive statistics for CD68+ cells.

	control	OTM
Minimum	2.829	5.658
25% Percentile	3.052	6.498
Median	4.823	9.554
75% Percentile	6.174	11.65
Maximum	6.257	12.17
Mean	4.683	9.233
Standard deviation	1.672	2.718
error of mean	0.8362	1.359
Lower 95% CI	2.022	4.908
Upper 95% CI	7.344	13.56

Table S10: Descriptive statistics for periodontal gap.

	distal		mesial	
	control	OTM	control	OTM
Minimum	0.06000	0.07000	0.1400	0.1300
25% Percentile	0.06000	0.07250	0.1400	0.1325
Median	0.06500	0.08500	0.1450	0.1450
75% Percentile	0.07750	0.09000	0.1575	0.1575
Maximum	0.08000	0.09000	0.1600	0.1600
Mean	0.06750	0.08250	0.1475	0.1450
Standard deviation	0.009574	0.009574	0.009574	0.01291
error of mean	0.004787	0.004787	0.004787	0.006455
Lower 95% CI	0.05227	0.06727	0.1323	0.1245
Upper 95% CI	0.08273	0.09773	0.1627	0.1655

Table S11: Descriptive statistics for periodontal bone loss.

	distal		mesial	
	control	OTM	control	OTM
Minimum	0.2300	0.3300	0.4000	0.3500
25% Percentile	0.2325	0.3300	0.4000	0.3525
Median	0.2800	0.3650	0.4050	0.3600
75% Percentile	0.3575	0.4150	0.4325	0.3750
Maximum	0.3700	0.4200	0.4400	0.3800
Mean	0.2900	0.3700	0.4125	0.3625
Standard deviation	0.06683	0.04690	0.01893	0.01258
error of mean	0.03342	0.02345	0.009465	0.006292
Lower 95% CI	0.1837	0.2954	0.3824	0.3425
Upper 95% CI	0.3963	0.4446	0.4426	0.3825

Table S12: Descriptive statistics for inclination.

	control	OTM
Minimum	76.77	72.49
25% Percentile	78.10	74.45
Median	82.31	80.70
75% Percentile	86.25	81.84
Maximum	87.49	82.10
Mean	82.22	79.00
Standard deviation	4.381	4.398
error of mean	2.190	2.199
Lower 95% CI	75.25	72.00
Upper 95% CI	89.19	85.99

Table S13: Descriptive statistics for distance between the first and second molar.

	control	OTM
Minimum	0.000	0.1100
25% Percentile	0.000	0.1200
Median	0.000	0.1600
75% Percentile	0.000	0.1700
Maximum	0.000	0.1700
Mean	0.000	0.1500
Standard deviation	0.000	0.02828
error of mean	0.000	0.01414
Lower 95% CI	0.000	0.1050
Upper 95% CI	0.000	0.1950
